# The Role of Carotenogenic Metabolic Flux in Carotenoid Accumulation and Chromoplast Differentiation: Lessons From the Melon Fruit

**DOI:** 10.3389/fpls.2019.01250

**Published:** 2019-10-30

**Authors:** Ari Feder, Noam Chayut, Amit Gur, Zohar Freiman, Galil Tzuri, Ayala Meir, Uzi Saar, Shachar Ohali, Fabian Baumkoler, Amit Gal-On, Yula Shnaider, Dalia Wolf, Nurit Katzir, Ari Schaffer, Joseph Burger, Li Li, Yaakov Tadmor

**Affiliations:** ^1^Newe Ya’ar Research Center, Agricultural Research Organization, Ramat Yishay, Israel; ^2^Germplasm Resource Unit, John Innes Center, Norwich, United Kingdom; ^3^Agricultural Research Organization, Volcani Center, Rishon LeZion, Israel; ^4^Robert W. Holley Center for Agriculture and Health, USDA-ARS, Cornell University, Ithaca, NY, United States

**Keywords:** carotenoids accumulation, OR genes, melon (*Cucumis melo* L.), tomato (*Solanum lycopersicum*), metabolic flux

## Abstract

Carotenoids have various roles in plant physiology. Plant carotenoids are synthesized in plastids and are highly abundant in the chromoplasts of ripening fleshy fruits. Considerable research efforts have been devoted to elucidating mechanisms that regulate carotenoid biosynthesis, yet, little is known about the mechanism that triggers storage capacity, mainly through chromoplast differentiation. The *Orange* gene (*OR*) product stabilizes phytoene synthase protein (PSY) and triggers chromoplast differentiation. *OR* underlies carotenoid accumulation in orange cauliflower and melon. The *OR*’s ‘golden SNP’, found in melon, alters the highly evolutionary conserved Arginine^108^ to Histidine and controls β-carotene accumulation in melon fruit, in a mechanism yet to be elucidated. We have recently shown that similar carotenogenic metabolic flux is active in non-orange and orange melon fruit. This flux probably leads to carotenoid turnover but known carotenoid turnover products are not detected in non-orange fruit. Arrest of this metabolic flux, using chemical inhibitors or mutations, induces carotenoid accumulation and biogenesis of chromoplasts, regardless of the allelic state of *OR*. We suggest that the ‘golden SNP’ induces β-carotene accumulation probably by negatively affecting the capacity to synthesize downstream compounds. The accumulation of carotenoids induces chromoplast biogenesis through a metabolite-induced mechanism. Carotenogenic turnover flux can occur in non-photosynthetic tissues, which do not accumulate carotenoids. Arrest of this flux by the ‘golden SNP’ or other flux-arrest mutations is a potential tool for the biofortification of agricultural products with carotenoids.

## Introduction

Carotenoids exhibit diverse roles during the plant life cycle, are essential components of the photosynthetic apparatus, color agents, and precursors of hormones and aroma compounds, and are also signaling molecules involved in various developmental and environmental signaling pathways (Nisar et al., 2015; Rodriguez-Concepcion et al., 2018a). Carotenoids are synthesized in plastids from the methylerythritol phosphate (MEP) pathway, followed by the biosynthesis of geranylgeranyl diphosphate (GGPP), a precursor of several essential isoprenoid pathways, including carotenoids (Ruiz-Sola et al., 2016). The first committed step in carotenogenesis requires the condensation of two GGPP molecules into phytoene, in an enzymatic reaction catalyzed by phytoene synthase (PSY), who’s activity divergence exists among its isozymes (Cao et al., 2019). Phytoene biosynthesis is followed by four desaturation reactions catalyzed by phytoene desaturase (PDS) and ζ-carotene desaturase (ZDS), to introduce *cis* double bonds, and isomerization to trans-configuration through ζ-carotene isomerase (ZISO) and carotene isomerase (CRTISO), producing all-*trans*-lycopene. Lycopene undergoes two cyclization steps during biosynthesis of either β-carotene (two β rings) or α-carotene (ε and β rings), converted by lycopene β-cyclase (CRTL-B) and lycopene ε-cyclase (CRTL-E) (Hirschberg, 2001; Alagoz et al., 2018). Carotenes undergo hydroxylation and epoxidation to form xanthophylls. Apocarotenoids, carotenoid-derived compounds, consist of volatile phytohormones ABA and strigolactones. Due to their critical role during the plant life cycle, carotenoids are subjected to multiple layers of regulation, affecting their biosynthesis, turnover, channeling and sequestration, including regulation of plastid development, transcriptional regulation of carotenogenic, as well as non-carotenogenic gene expression and post-transcriptional regulation, all of which determine the final composition and abundance of carotenoids.

Carotenoids are highly abundant in the chromoplasts and accumulate mainly in flowers, fruits, and vegetables (Yuan et al., 2015b; Sun et al., 2018). Chromoplasts can differentiate from different plastids, and exhibit considerable variance in structure and biochemical composition, all of which are highly enriched in sequestration substructures, enabling high rates of biosynthesis and stable storage capacities (Sun et al., 2018). As such, chromoplast formation during fruit ripening is one of the main factors governing fruit pigmentation, nutritional value and taste. Chromoplast biogenesis involves a matrix of interactions, including hormonal signaling, gene expression and post-transcriptional modifications (Harris and Spurr, 1969; Egea et al., 2010; Li and Yuan, 2013).

Melon’s, *Cucumis melo* L. (*Cucurbitaceae*), fruit flesh color appears either as white, green or orange (Burger et al., 2009). Green- and white-flesh melon fruit contain negligible amounts of carotenoids, whilst orange-flesh melon accumulates massive amount of β-carotene. Carotenoid accumulation in melon fruit is conferred by the dominant allele at the *gf* (*green flesh*) locus (Hughes, 1948; Clayberg, 1992). The melon’s *ORANGE* gene (*CmOR*) resides at the *gf* locus and its ‘golden’ SNP (single nucleotide polymorphism), replacing the evolutionary conserved Arginine^108^ (Arg^108^) with Histidine (His), controls chromoplast differentiation and carotenoid accumulation during melon fruit development (Tzuri et al., 2015). Except for this single amino-acid substitution, not much is known about the initial molecular signaling pathway triggering chromoplast differentiation. In this mini-review, we emphasize the role of the carotenogenic metabolic flux, its association with the ‘golden SNP’ and its arrest during the process of fruit ripening, which we suggest initiates chromoplast differentiation and carotenoid accumulation.

## Regulation of Carotenoid Biosynthesis

Regulation of carotenoid biosynthesis and storage in non-photosynthetic tissues has drawn much attention in recent decades (Giuliano, 2017). Most of our knowledge about the regulation of the carotenoid pathway during fruit development stems from tomato, *Solanum lycopersicum* L. (*Solanaceae*), which serves as a model plant for the study of climacteric fleshy fruit development. The main carotenoid accumulated during tomato fruit ripening is lycopene. This accumulation, one of many ripening-related modifications, is governed by a transcriptional network, which regulates the ethylene burst, results in chromatin modification, and underlies the massive transcriptional modifications during fruit ripening (Oeller et al., 1991; Alba et al., 2005; Zhong et al., 2013; Giovannoni et al., 2017; Li et al., 2019). At the carotenogenic transcriptional level, lycopene upstream coding genes, including *PSY*1, *PDS*, and *CRTISO*, are transcriptionally upregulated, while the lycopene cyclases, *CRTL-B* and *CRTL-E*, are downregulated, enabling lycopene accumulation (Ronen et al., 1999; Isaacson et al., 2002). Among these processes, the transcriptional activation of *PSY*1 is one of the major flux-controlling factors during carotenoid accumulation in ripening fruit (Fraser et al., 1994; Fraser et al., 2002). In contrast to tomato, carotenoid accumulation during melon fruit ripening does not depend on the ethylene climacteric burst. Moreover, inhibition of ethylene biosynthesis does not affect fruit carotenoid accumulation in ‘Védrantais’ melon fruit (Ayub et al., 1996; Pech et al., 2008). These observations indicate two divergent regulating mechanisms of carotenoid accumulation in ripening tomato and melon fruits, consistent with recent findings indicating that different transcriptional networks regulate ethylene signaling in these species (Lu et al., 2018). As in tomatoes, carotenoid accumulation during melon fruit ripening is associated with the transcriptional upregulation of β-carotene upstream carotenogenic structural genes (*PSY*1, *PDS*, and *ZDS*), along with an increase in PSY1 protein abundance (Chayut et al., 2015). However, these transcriptional changes are not sufficient to induce carotenoid accumulation in melon, as similar patterns of carotenogenic gene expression along with PSY1 protein abundance have been found in both β-carotene-accumulating and non-accumulating melon accessions, similar to that found in cauliflower (*Brassica oleracea*) (Li et al., 2006; Chayut et al., 2015; Chayut et al., 2017). Enzymatic carotenoid’s turn over to apocarotenoids is regulated by carotenoid cleavage dioxygenases (CCDs). A comparison between melon fruit flesh of carotenoid accumulating versus non-accumulating accessions correlates apocarotenoid with carotenoid accumulation, while both orange and non-orange fruit show elevated *CmCCD1* transcription during fruit ripening, suggesting substrate availability to be the limiting factor in apocarotenoid production in melon (Ibdah et al., 2006).

Several genes have been reported as plastid size and development regulators, involving different signaling pathways (Levin et al., 2006; Galpaz et al., 2008) but, to date, the only gene which has been found to directly regulate chromoplast biogenesis is the *OR* gene. *OR* belongs to a DnaJ-like family termed DnaJ-E, a non-canonical DnaJ-related protein coding gene family, coding for cysteine-rich zinc finger domain proteins, which have undergone vast duplication in plants during evolution. *OR* and several other members of the family possess plastid-related, protein-assembly factor activity (Pulido and Leister, 2018). *OR* was first described in cauliflower, as a single semi-dominant gene mutation conferring orange-colored curds (Crisp et al., 1975), it triggers chromoplast differentiation associated with high levels of β-carotene accumulation (Li et al., 2001; Paolillo et al., 2004). Positional cloning identified the causative mutation as an insertion of a *copia* element in the protein coding region of the *BoOR* gene, which leads to various altered transcripts and to the orange curd phenotype. The *OR* gene is highly conserved in the plant kingdom, and also found in algae, but not in photosynthetic bacteria or non-photosynthetic species (Lu et al., 2006). Ectopic overexpression (OE) of the single-cell green alga *Chlamydomonas reinhardtii* *OR* gene enhances carotenoid content (Morikawa et al., 2018).

The *Arabidopsis* genome harbors two homologs of the *OR* gene, termed *AtOR* and *AtOR-like* (Zhou et al., 2015). Both interact with PSY in *Nicotiana benthamiana* leaf chloroplasts, are physically associated with PSY *in-vitro*, and are needed to stabilize PSY protein levels, activity, and carotenoid biosynthesis, as observed in *AtOR/AtOR-like* double knockout. The OR protein consists of two domains: the N-terminus binds PSY, protecting it from Clp-mediated proteolysis (Zhou et al., 2015; D’Andrea et al., 2018; Rodriguez-Concepcion et al., 2018b; Welsch et al., 2018). These findings, indicate that the DnaJ-like proteins have maintained the original function of canonical DnaJ proteins, acting as chaperones, synergistically determining protein turnover and quality along with proteases (Bukau et al., 2006). The C-terminus contains two putative transmembrane domains, along with the cysteine-rich ZF domain, which has been found to mediate OR protein dimerization (Zhou et al., 2015).

In melon, the *CmOR* gene was found to reside at the *gf (green flesh)* locus, regulating β-carotene accumulation in the fruit mesocarp. *CmOR* exhibits two major haplotypes differentially associated with β-carotene accumulation within a population of 49 melon accessions. A single SNP, termed the ‘golden SNP’, replaces a conserved amino acid Arg^108^ with His, in *OR*, underlies the trait (Tzuri et al., 2015). Recently, genome-wide, linkage-disequilibrium mapping of 177 melon accessions re-confirmed the causality of the ‘golden SNP’ (Gur et al., 2017). In the mesocarp of the *CmOR* knockout mutant, small amounts of carotenoids are accumulated in late fruit developmental stages associated with an increase in PSY abundance, which is related to an increase in transcriptional abundance of *CmOR-like* (Chayut et al., 2017).

Overexpression of *AtOR* *^His^* significantly increases carotenoid accumulation in dark-grown, seed-derived *Arabidopsis* calli, as compared with *AtOR* *^Arg^* (Tzuri et al., 2015; Yuan et al., 2015a). Similar to *BoOr* *^Mut^* *, AtOR* *^His^* triggers membranous chromoplast biogenesis, while *AtOR* *^Arg^* does not (Paolillo et al., 2004; Yuan et al., 2015a). Both alleles of *CmOR* and *AtOR* (His and Arg) are equally capable of binding and stabilizing PSY protein levels, a characteristic governed by the N-terminus of the OR protein. The ‘golden SNP’ governs an additional role of *OR* gene, regulating carotenoid accumulation and plastid formation in melon fruit. This function in melon, can be introduced to other plant systems (Yuan et al., 2015a; Chayut et al., 2017; Yazdani et al., 2019). Ectopic overexpression of *AtOR* and *AtOr* *^His^* in tomatoes has been shown to have a minimal effect on photosynthetic tissues, similar to OE in *Arabidopsis* plants or the effect of natural variation in *OR* ‘golden SNP’ on melon plant performance. However, tomato flowers and fruit are dramatically affected by the ‘golden SNP’; carotenoid abundance in flower petals increases by more than two-fold in *AtOR* *^His^* OE lines, mainly due to an increase in β-carotene, but it is not affected by *AtOR* OE (Yazdani et al., 2019). During early fruit developmental stages, *AtOR* *^His^* shows a significant increase in carotenoid accumulation, dominated by elevation of β-carotene and associated with chromoplast differentiation, while *AtOR* only has a minor effect on carotenoid content and composition. During fruit ripening, the climacteric ethylene induces lycopene accumulation in *AtOR* and *AtOR* *^His^* OE, as well as in the control, an M82-type processing tomato cultivar. Nevertheless, the effect of *AtOR* *^His^* OE on carotenoid content is much more dramatic; a 250% increase in lycopene and 300% in β-carotene compared to the control, with *AtOR* OE exhibiting only a 25% increase in lycopene content (Yazdani et al., 2019). The effect of *AtOR* *^His^* OE during the pre-climacteric green stages of tomato fruit development, i.e., ethylene-independent β-carotene accumulation associated with chromoplast differentiation, exhibits a striking similarity to the effect of *CmOR* *^His^* during melon fruit development.

The increase in carotenoid accumulation during ripening stages of the OE lines suggests that *OR* also serves as a limiting factor in carotenoid accumulation during tomato fruit ripening; this is further supported by the transcriptional upregulation of both *SlOR* and *SlOR-like* during fruit ripening (Tomato Expression Atlas database: http://tea.solgenomics.net) (Fernandez-Pozo et al., 2017). These studies strongly suggest a conserved function of the *OR* and *OR-like* genes.

**Figure 1:**
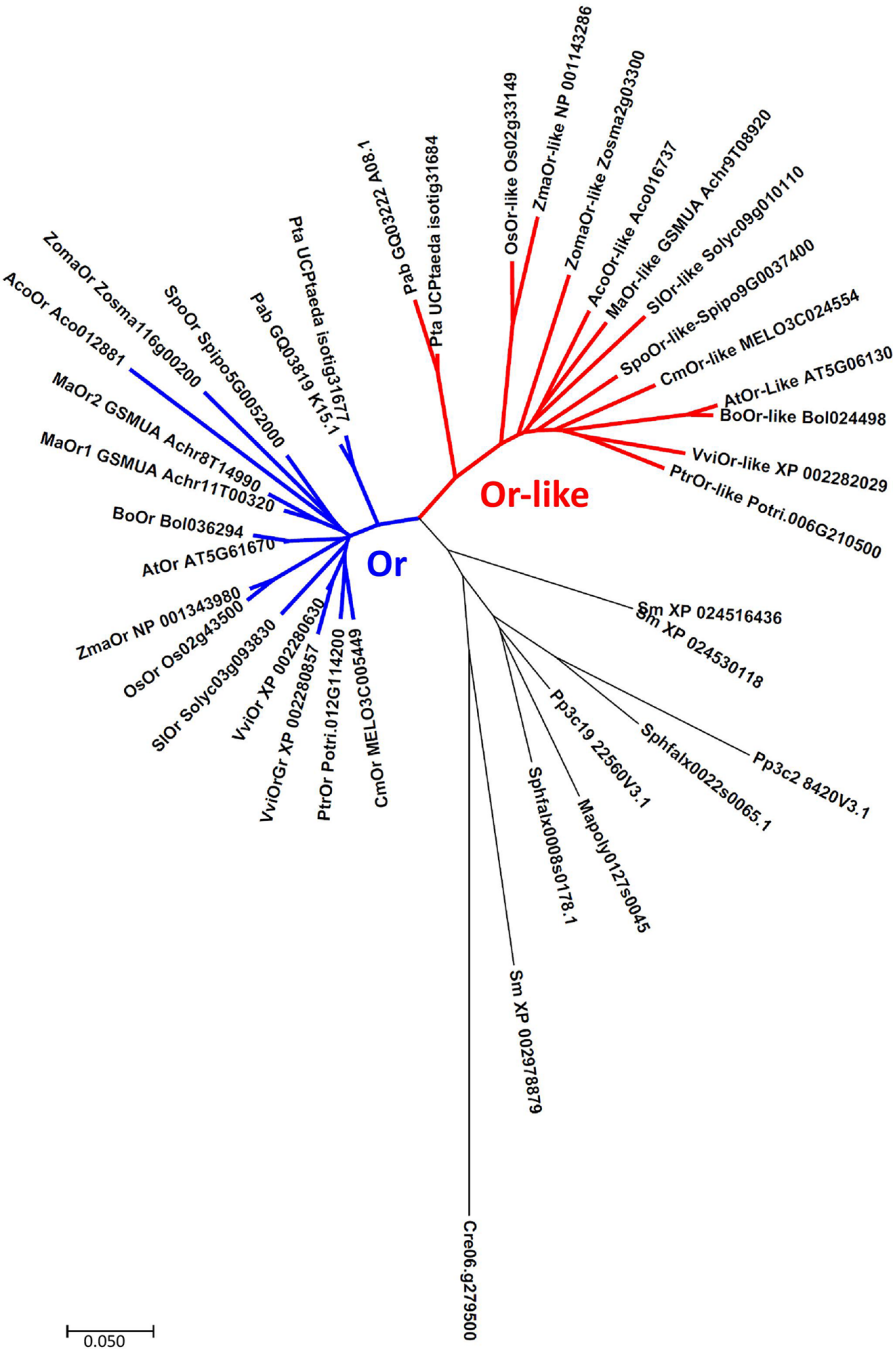
Neighbor-joining phylogenetic tree of OR and OR-like proteins. Cluster analysis associate’s gene duplication with bryophytes and exhibits two distinctively separate clades, conserved from gymnosperms onwards. Abbreviations- Algae: Cre (*Chlamydomonas reinhardtii*). Liverworts: Mapoly (*Marchantia polymorpha*). Mosses: Pp (*Physcomitrella patens*), Sphfalx (*Sphagnum fallax*). Lycophytes: Sm (*Selaginella moellendorffii*). Gymnosperms: Pte (*Pinus taeda*), Pab (*Picea abies*). Angiosperms: Aco (*Ananas comosus*), Ma (*Musa acuminata*), Spo (*Spirodela polyrhiza*), Zoma (*Zostera marina*), Os (*Oryza sativa*), Zma (*Zea mays*), Sl (*Solanum lycopersicum*), Vvi (*Vitis vinifera*), Ptr (*Populus trichocarpa*), At (*Arabidopsis thaliana*), Bo (*Brassica oleracea*), Cm (*Cucumis melo*). Sequences were downloaded from Phytozome (http://www.phytozome.net), National Center for Biotechnology Information (http://www.ncbi.nlm.nih.gov), TAIR (https://www.arabidopsis.org), Sol Genomics Network (https://solgenomics.net), CuGenDB (http://cucurbitgenomics.org), and ConGenIE (http://congenie.org). Created with MEGA 7 (Kumar et al., 2016).

More insight into the relationship between *OR/OR-like* and chromoplast development can be gained from a phylogenetic analysis (**Figure 1**). The hierarchical clustering of published *OR* and *OR-like* sequences indicates that lower plant evolution is generally associated with duplication of the *OR* gene, while clear differentiation into two *OR* and *OR-like* distinct clades is highly conserved from gymnosperms onwards, indicating a different unique function of each protein, in addition to their common PSY binding activity (Zhou et al., 2015). Although chromoplasts are mainly associated with angiosperm flowers and fruit, examples of similar tubular structures that are frequently present in fruit can be found in ripe red seeds of cycads (Whatley, 1985). This associates OR/*OR-like* evolutionary specialization with the early evolution of seed-producing plants, designating the vast majority of agricultural crops as candidates for biofortification through *OR*-based modification.

The difference between climacteric and *OR* *^His^*-dependent, non-climacteric fruit carotenoid accumulation, demonstrated here by tomato and melon, suggest distinct regulating mechanisms of fruit-carotenoid accumulation, which can stand alone or complement each other. The major factor in climacteric-regulated carotenoid accumulation is the developmentally associated transcriptional changes in carotenogenic gene expression regulated by ethylene (Alba et al., 2005). The *OR* *^His^*-dependent carotenoid accumulation mechanism is probably associated with post-translational regulation of the carotenogenic metabolic flux, which will be discussed in the next section.

## Controlling the Carotenoid Metabolic Flux and Accumulation

The increase in *PSY*1 activity during fruit ripening is a major factor that limits lycopene accumulation in tomatoes (Cao et al., 2019). This increased activity, serves as an example of a direct relationship between carotenogenic gene activity and carotenoid accumulation. However, an immature green tomato does not accumulate significant carotenoid levels, even though it exhibits higher enzymatic activities of PSY, PDS and CRTL-B compared to ripening fruit, irrespective of the dramatic increase in β-carotene and lycopene abundance during fruit ripening (Fraser et al., 1994). The increase in carotenogenic activity during immature fruit stages is attributed to actively photosynthetic tissue resulting in a high rate of carotenoid turnover (Lytovchenko et al., 2011; Brazel and O’Maoileidigh, 2019). This indicates that intensive carotenogenic flux during fruit development does not necessarily relate to carotenoid accumulation, raising questions about the mechanism mediating *OR* *^His^*-dependent carotenoid accumulation and chromoplast biogenesis. Additionally, this indicates that turnover of carotenoids creates a metabolic flux that could be utilized for carotenoid accumulation if combined with *OR* *^His^* (Yazdani et al., 2019).

Several reports have documented that changes in the carotenoid biochemical flux by itself, without any additional developmental program, could be sufficient to trigger carotenoid accumulation. Over-expression of *PSY1* in tomato results in chromoplast-like plastids in premature fruit, independent of the ripening program, and exhibit a metabolite-induced plastid transition (Fraser et al., 2007). Over-expression of *PSY1* in *Arabidopsis* increases the PSY protein level, leading to increased carotenoid (β-carotene) levels in dark-grown, seed-derived calli but not in leaves (Maass et al., 2009). In addition, overexpression of crtB, a bacterial PSY, in white carrots, result in a similar increase in carotenoids deposited as crystals, indicating that sequestration into crystals can be achieved through an increase of the pathway flux (Maass et al., 2009).

Cauliflower calli containing either *BoOR* *^WT^* or *BoOR* *^Mut^* produce a similar pattern of phytoene accumulation following treatment with norflurazon (NF, a PDS inhibitor), indicating that the carotenoid metabolism flux is similarly active in both calli, even though only *BoOR* *^Mut^* induces carotenoid accumulation (Li et al., 2006). In melon, the natural *CmOR* genetic variation is based on the ‘golden SNP’ (Tzuri et al., 2015). Carotenogenic gene expression and the carotenoid metabolic flux are similarly active in orange and non-orange melons (Chayut et al., 2017). Ethyl methanesulfonate (EMS)-induced mutagenesis of CEZ, an orange-flesh Charentais-type melon, generated an additional allele of *CmOR*, a nonsense allele called ‘*low-β’*. The *CmOR* ‘*low*-*β*’ allele completely lacks the CmOR protein, resulting in a low PSY1 protein level and low carotenoid biosynthesis metabolic flux, as revealed by PSY1 western blot analysis and the reduced accumulation of phytoene after treatment with NF (Chayut et al., 2017).

An additional mutant isolated from the CEZ mutation library, called “*Yellow-Orange Flesh I*’ (*yofi*), exhibits a yellow-orange flesh color due to a nonsense mutation in *CRTISO*, stopping the carotenogenic metabolic flux in prolycopene (*tetra*-*cis*-lycopene), since the isomerized product, *all*-*trans*-lycopene, is a necessary precursor of the β-cyclase enzymes (Galpaz et al., 2013). *Yofi* was a parental line of two segregating F_2_ populations. The first population was generated from a cross between *yofi* and the green-fleshed inbred line ‘Noy Yizre’el’, harboring the *CmOR* *^Arg^* allele, generating a population that segregates to *CmOR* *^Arg^* */CmOR* *^His^* and *CRTISO/crtiso*, wild type and nonsense (*yofi*) alleles. The second population originated from a cross between *yofi* and ‘*low*-β’, which segregated to *CmOr* *^His^* */’low* β’ in addition to *CRTISO/crtiso* alleles. High Performance Liquid Chromatography (HPLC) carotenoid analysis of F_2_ segregants selected for defined genotypes revealed that, as expected, *CmOR* *^His^* segregants accumulate β-carotene in the *CRTISO* background and pro-lycopene in the *yofi* background. Interestingly, *CmOR* *^Arg^* and ‘*low-β*’ segregants, which harbor the *yofi* allele, accumulate prolycopene, similar to *CmOR* *^His^* */yofi* segregants. This indicates that arrest of the carotenogenic metabolic flux can induce carotenoid accumulation and chromoplast formation regardless of *CmOR* allelic variation. In addition, it indicates that a low metabolic flux, as observed in ‘*low-β’*, is sufficient to induce carotenoid accumulation and chromoplast formation, similar to a high metabolic flux, as observed in the *CmOR* natural variation, when the flux is blocked. This suggests that the ‘golden SNP’ stabilizes β-carotene by inhibiting beta-ring hydroxylase activity (Chayut et al., 2017). Moreover, these results prompted us to suggest an arrest of the carotenogenic metabolic flux, by mutations or by genome editing, as an optional strategy to induce carotenoid accumulation in non-carotenoid accumulating tissues.

## Conclusions and Future Perspectives

This mini-review summarizes studies that indicate the role of the *OR* gene in stabilizing the carotenogenic metabolic flux for chromoplast differentiation and carotenoid accumulation.

Selected studies, presented here, suggest that the *OR* ‘golden SNP’ mediates storage capacity in melon fruit, and in other non-photosynthetic tissues, by arresting the carotenogenic metabolic flux downstream to β-carotene, leading to chromoplast biogenesis and carotenoid accumulation, mainly β-carotene. This suggests an arrest of the carotenogenic metabolic flux in plant tissues, which normally do not accumulate carotenoids, as a potential biofortification tool. In the age of CRISPR-mediated genome editing, introgression of the ‘golden SNP’ in horticultural crops should prove an efficient tool for the development of novel products of high nutritional value, and for further elucidating the mechanism of chromoplast biogenesis.

The close relationship between *OR* and *OR-like*, and hints to a partially complementary function of *OR-like*, should be looked at more closely to gain further insight into the biogenesis and evolution of chromoplasts.

Carotenoid accumulation in tomatoes and in other fruit is regulated by a climacteric ethylene burst. In melon, fruit-flesh carotenoid accumulation depends on the ‘golden SNP’, independent of ethylene regulation. Combining these polyphyletic regulation processes may well enhance attempts to break the limits of carotenoid biofortification of agricultural products.

## Author Contributions

All authors contributed to the OR gene work in melon, *Arabidopsis* and tomato. All authors contributed to the writing of this mini review.

## Conflict of Interest

The authors declare that the research was conducted in the absence of any commercial or financial relationships that could be construed as a potential conflict of interest.
